# High Prevalence of Post-stroke Anxiety in Elderly Patients Following COVID-19 Outbreak

**DOI:** 10.3389/fpsyt.2021.699869

**Published:** 2021-06-24

**Authors:** Meiling Yao, Hongjie Li, Ying Luo, Ling Li, Jian Yu

**Affiliations:** Department of Neurology, The First Affiliated Hospital, Sun Yat-Sen University, Guangdong Provincial Key Laboratory of Diagnosis and Treatment of Major Neurological Diseases, National Key Clinical Department and Key Discipline of Neurology, Guangzhou, China

**Keywords:** post-stroke anxiety, elderly patients, acute ischemic stroke, risk factors, COVID-19

## Abstract

**Objective:** Post-stroke anxiety (PSA) is a common affective disorder in patients with ischemic stroke. The elderly are more susceptible to mental health issues, however, few studies have so far focused on PSA in elderly patients, especially in the context of the COVID-19, causing psychological issues in the general population. The aim of the present study was to assess the prevalence and risk factors of PSA in elderly patients following COVID-19 outbreak.

**Methods:** We retrospectively analyzed 206 elderly inpatients with newly diagnosed acute ischemic stroke in the First Affiliated Hospital, Sun Yat-sen University, from January 2020 to December 2020. Patients were categorized into the PSA group and the non-PSA group based on Hamilton Anxiety Scale scores at admission (within 1 week after stroke onset). Demographic and clinical data, mental state by Mini-Mental State Examination, depression by Hamilton Depression Scales (HAMD), and stroke severity and outcome by National Institutes of Health Stroke Scale (NIHSS) and modified Rankin Scale were compared between the two groups. Univariate analysis and binary logistic regression analysis were used to analyze risk factors associated with PSA. We determined the cutoff scores for significant predictors of PSA using the area under the curve (AUC) and receiver operating characteristic.

**Results:** Of the 206 stroke patients, 62 (30.1%) developed anxiety. Binary logistic regression analysis showed that female gender [adjusted odds ratio (aOR): 2.288, 95% confidence interval (CI):1.021–5.128, *P* = 0.044], high NIHSS scores [aOR: 1.264, 95% CI: 1.074–1.486, *P* = 0.005] and HAMD scores [aOR: 1.345, 95% CI: 1.215–1.490, *P* < 0.001] were independent risk factors for PSA. The cutoff threshold for the NIHSS scores was 3.5 points with an AUC of 0.64 and the cutoff threshold for HAMD scores was 5.5 points with an AUC of 0.89.

**Conclusion:** Our results showed a high incidence of PSA in elderly patients after the COVID-19 outbreak. Female gender, high NIHSS and HAMD scores were the independent risk factors for PSA.

## Introduction

Acute ischemic stroke is one of the leading causes of death and disability worldwide and has become a major disease burden for elderly patients in China (1, 2). Many studies have shown that anxiety and depression can affect patient's quality of life (3, 4). Anxiety and depression are both common complications in stroke patients, but post-stroke anxiety (PSA) appears to have a more stable and lasting effect than post-stroke depression (5–7). It has been reported that PSA occurs in 1.8–27% of stroke survivors (5, 8–12), however, the prevalence of PSA in elderly patients remains unclear.

Since December 2019, the COVID-19 pandemic has caused extensive anxiety and psychological issues in the general public and healthcare workers (13, 14). Although COVID-19 has been effectively controlled in China, it is still spreading around the world, and sporadic cases occur in some places in China. With the stricter management in hospitalized patients including limitations on patient visitations from family members, the elderly patients are more prone to feel loneliness and insecurity, leading to emotional issues including anxiety (15, 16). There is no data so far on the prevalence of PSA in elderly inpatients in the context of COVID-19. The present study aimed to fill this knowledge gap and explore independent risk factors for PSA.

## Patients and Methods

### Patients

This study was approved by the ethics committee of the First Affiliated Hospital of Sun Yat-sen University. The study was conducted in accordance with the Declaration of Helsinki. Consecutive inpatient paper medical records and electronic medical records from January 2020 to December 2020 were reviewed at the Department of Neurology, the First Affiliated Hospital of Sun Yat-sen University. After a detailed evaluation of the inclusion and exclusion criteria, 206 patients were included in this study. The inclusion criteria were: (1) Patients were diagnosed with ischemic stroke according to the International Classification of Diseases (ICD-10) (17), with infarction sites confirmed by brain CT or MRI. (2) Patients were admitted to the hospital within 1 week after ischemic stroke onset. (3) Patient age was ≥ 60 years old. The exclusion criteria were: (1) Previous diagnosis of anxiety, depression, and other mental disorders. (2) Patients with severe aphasia, confusion, or patients unable to complete the relevant scale tests. (3) Presence of other diseases which can cause emotional disturbance, including severe heart failure, thyroid diseases, severe liver or kidney dysfunction.

### Measures

At admission, sociodemographic characteristics (gender, age, marital status, and education) and risk factors for cerebrovascular disease (diabetes mellitus, hypertension, smoking, and drinking) were recorded. Patients were etiologically classified according to the Trial of Org 10172 in Acute Stroke Treatment (TOAST) classification system (18).

The Hamilton Anxiety Scale (HAMA) was employed by two experienced neurologists to evaluate the anxiety of patients. Patients with HAMA scores ≥7 were considered to experience anxiety and were enrolled in the PSA group (19, 20). Patients with HAMA scores <7 were considered not to exhibit anxiety and therefore classified in the non-PSA group. HAMA can be summarized into two types of factor structure: one is psychological symptoms consisting of anxious mood, tension, fears, insomnia, cognitive changes, depression, and behavior at interview, while the other is somatic symptoms including muscular, sensory, gastrointestinal, genitourinary, respiratory, cardiovascular and autonomic symptoms (21). Depression was assessed using the Hamilton Depression Scales (HAMD). Meanwhile, the National Institutes of Health Stroke Scale (NIHSS) and the modified Rankin Scale (mRS) were used to evaluate stroke severity and outcome while the Mini-Mental State Examination (MMSE) to assess cognitive functions.

### Statistical Analysis

All statistical analyses were performed using SPSS 24.0 (IBM Corp, Armonk, NY). Quantitative data with normal distribution were described as mean ± standard deviation (x ± s), while non-normally distributed quantitative data were expressed as median with 25 percentile and 75 percentiles. Qualitative data were described using frequencies and percentages. Univariate analysis of quantitative data were compared with the independent two-sample *t*-tests or Mann–Whitney *U*-tests. Pearson's Chi-square test or Fisher's exact test were used to compare different categories of qualitative variables. Significant features in univariate analysis were selected for multivariate binary logistic regression analysis. To determine the cutoff scores for independent predictors of PSA vs. non-PSA groups, we used the receiver operating characteristic (ROC) curve. The accuracy of significant predictors was determined with the area under the ROC curve (AUC). *P*-values lower than 0.05 were considered statistically significant.

## Results

### Sociodemographic and Clinical Characteristic

Two hundred and six eligible patients were included in this study. The median age of the patients was 67 (range 60–90), of which 60.7% were male. Twenty-three patients (11.2%) were divorced or widowed. Seventeen percent (17.5%) had received university education or above (Table 1). Eighty-four patients (59.2%) had a history of diabetes mellitus and 151 (73.3%) had hypertension. The rates of individuals reporting smoking and drinking were 44.2% and 26.2%, respectively. According to the TOAST etiology classification, 61.6% of patients were classified as large-artery atherosclerosis. In 37.4% of patients, the lesion site was in the right hemisphere.

**Table 1:** Sociodemographic and clinical variables between the PSA and non-PSA groups.

	**All patients**	**Post-stroke anxiety**		***P*-value**
	**(*n* = 206)**	**No (*n* = 144)**	**Yes (*n* = 62)**	
**Demographic variable**				
Gender (*n*, %)^†^				0.007^*^
Male	125 (60.7)	96 (66.7)	29 (46.8)	
Female	81 (39.3)	48 (33.3)	33 (53.2)	
Age(years)^#^	67 (64,74)	67 (64,73)	68 (63,74)	0.650
Marital status (*n*, %)^†^				0.014^*^
Married	183 (88.8)	133 (92.4)	50 (80.7)	
Single (divorced/widowed)	23 (11.2)	11 (7.6)	12 (19.3)	
Level of education (*n*, %)^†^				0.196
Primary school and below	87 (42.2)	56 (38.9)	31 (50.0)	
Secondary school	83 (40.3)	59 (41.0)	24 (38.7)	
University and above	36 (17.5)	29 (20.1)	7 (11.3)	
**Vascular risk factors**				
Diabetes mellitus (*n*, %)^†^	84 (59.2)	55 (38.2)	29 (46.8)	0.250
Hypertension (*n*, %)^†^	151 (73.3)	105 (72.9)	46 (74.2)	0.849
Smoking (*n*, %)^†^	91 (44.2)	67 (46.5)	24 (38.7)	0.300
Drinking (*n*, %)^†^	54 (26.2)	37 (25.4)	17 (27.4)	0.796
**TOAST classification (*****n*****, %)**				
Large-artery atherosclerosis	127 (61.6)	90 (70.9)	37 (29.1)	0.318
Cardio embolism	14 (6.8)	10 (71.4)	4 (28.6)	
Small-vessel occlusion	56 (27.2)	36 (64.3)	20 (35.7)	
Other determined etiology	2 (1.0)	1 (50.0)	1 (50.0)	
Undetermined etiology	7 (3.4)	7 (100.0)	0 (0)	
**Lesion location (*****n*****, %)**				
Left hemisphere	66 (32.0)	49 (74.2)	17 (25.8)	0.145
Right hemisphere	77 (37.4)	51 (66.2)	26 (33.8)	
Bilateral hemisphere	21 (10.2)	14 (14.7)	7 (6.3)	
Brainstem	32 (15.5)	20 (62.5)	12 (37.5)	
Cerebellum	10 (1.9)	10 (100)	0 (0)	
**Neurophysiological test scores**				
NIHSS^#^	4 (2,5)	3 (2,5)	4 (3,7)	0.001^*^
mRS^#^	2 (1,3)	2 (1,3)	2 (1,4)	0.002^*^
MMSE^#^	20 (15,24)	25 (21,28)	23 (17,26)	0.001^*^
HAMD^#^	4 (1,8)	3 (1,5)	10 (6,15)	0.000^*^

*PSA, post-stroke anxiety; NIHSS, National Institute of Health Stroke Scale; mRS, Modified Rankin Scale; MMSE, Mini-Mental State Examination; HAMD, Hamilton Depression Scale*.

† *n(%), Pearson's Chi-square test; n(%), Fisher's exact test*;

# *median(25%Q, 75%Q), Mann–Whitney U-tests*;

* *P < 0.05*.

**Figure 1:**
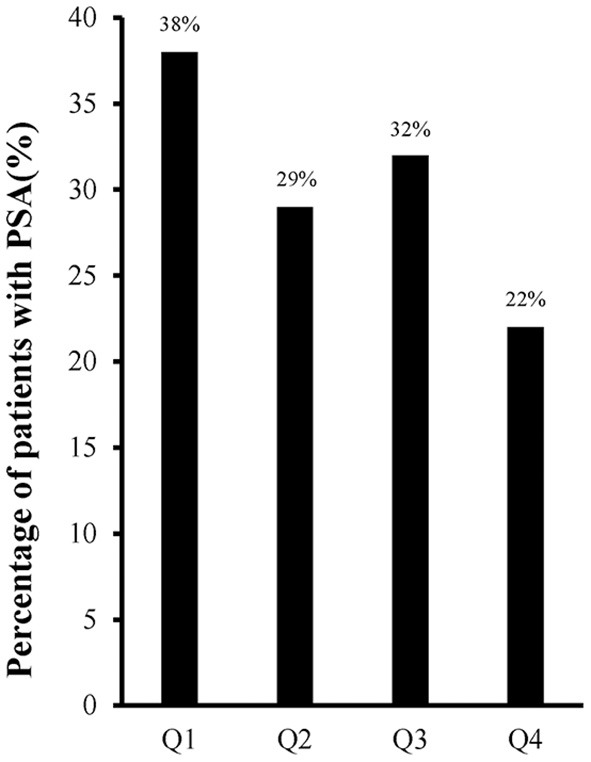
The incidence of PSA each quarter in 2020. Q1, the first quarter; Q2, the second quarter; Q3, the third quarter; Q4, the fourth quarter.

**Figure 2:**
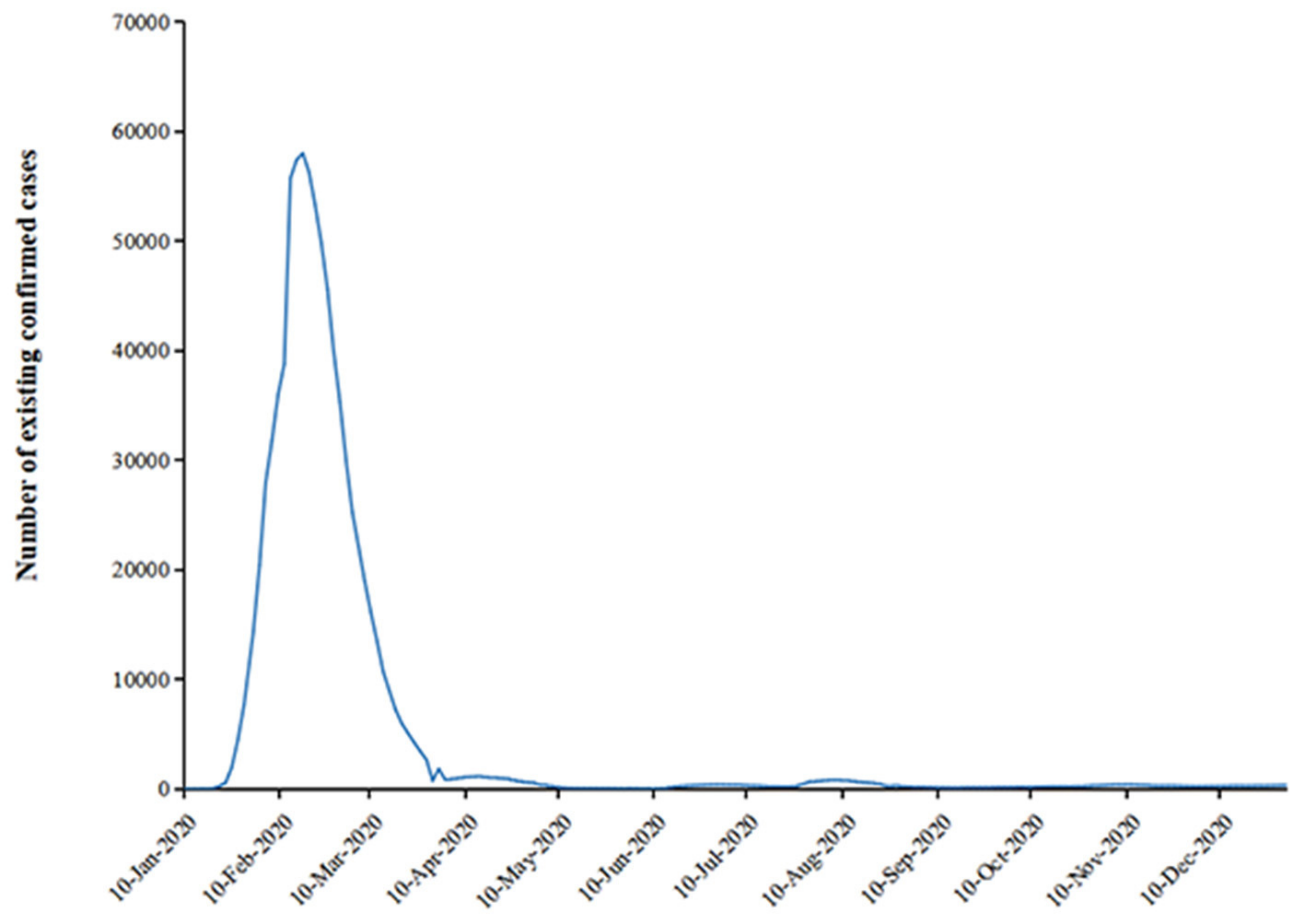
Trends in the number of existing patients with COVID-19 in China in 2020.

Sixty-two (30.1%) of the 206 elderly patients were diagnosed with PSA. According to official information released by the National Health Commission of the People's Republic of China (http://www.nhc.gov.cn/xcs/yqtb/list_gzbd.shtml), the COVID-19 pandemic in China has been effectively controlled by the end of the first quarter in 2020. We analyzed the incidence of PSA quarterly in 2020, and found that the incidence of PSA showed a downward trend after the COVID-19 pandemic was controlled (Figures 1, 2). Psychological symptoms of PSA were higher than somatic symptoms by HAMA scores. Psychological symptoms mainly manifested as cognitive changes (88%), insomnia (74.2%), anxious mood (72.6%) and tension (66.1%), while somatic symptoms as muscular symptoms (51.6%), sensory symptoms (58.1%), cardiovascular symptoms (40.3%) and autonomic symptoms (40.3%). Table 1 demonstrated a higher proportion of female gender and single status patients (divorced or widowed) in the PSA group. Patients with PSA were also more likely to exhibit higher mRS scores, higher scores of mRS, NIHSS and HAMD than patients without PSA.

### Factors Associated With PSA Among Patients

The following factors were assessed in this study: gender, age, marital status, level of education, vascular risk factors, stroke type, stroke lesion location, neurophysiological test scores. Results from the mono-factorial analysis showed that female gender (*P* = 0.007), single status (including being divorced or widowed) (*P* = 0.014), MMSE scores (*P* = 0.001), mRS scores (*P* = 0.002), NIHSS scores (*P* = 0.001), and HAMD scores (*P* < 0.001) were significantly associated with PSA. There were no statistically significant differences for any of the other examined factors (Table 1).

All risk factors with significant associations found in the univariate analysis were then included as predictive indicators in binary logistic regression. Multivariable analysis indicated that female gender (adjusted odds ratio (aOR): 2.288, 95% confidence interval (CI): 1.021–5.128; *P* = 0.044), NIHSS scores (aOR: 1.264 per one-point increase in NIHSS scores; 95% CI: 1.074–1.486; *P* = 0.005) and HAMD scores (aOR: 1.345 per one point increased in HAMD score; 95% CI: 1.215–1.490; *P* < 0.001) were independent predictors of PSA (Table 2).

**Table 2:** Multivariate logistic regression analysis for identification of factors associated with PSA in elderly patients.

**Variable**	**B**	**SE**	**Wald**	**aOR**	**95%CI**	***P***
Gender (female vs. male)	0.828	0.412	4.038	2.288	1.021–5.128	0.044^*^
Marital status (single vs. married)	0.301	0.647	0.217	1.351	0.381–4.800	0.641
NIHSS score	0.234	0.083	7.991	1.264	1.074–1.486	0.005^*^
mRs score	−0.205	0.210	0.953	0.815	0.540–1.229	0.329
MMSE score	−0.048	0.035	1.913	0.167	0.890–1.020	0.167
HAMD score	0.297	0.052	32.34	1.345	1.215–1.490	0.000^*^

*PSA, post-stroke anxiety; NIHSS, National Institute of Health Stroke Scale; mRS, Modified Rankin Scale; MMSE, National Institute of Health Stroke Scale; HAMD, Hamilton Depression Scales*.

*B, regression coefficient; SE, standard error; aOR, adjusted odds ratio; CI, confidence interval*;

* *P < 0.05*.

**Figure 3:**
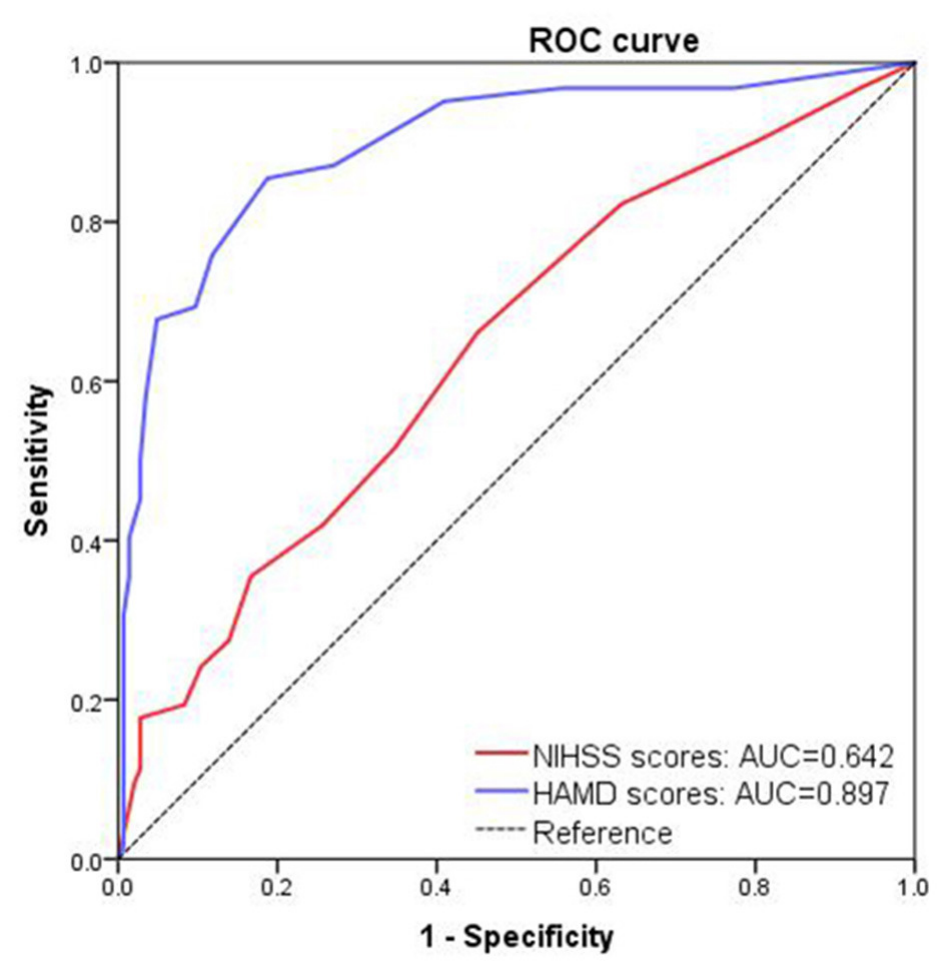
Receiver operating characteristic (ROC) curve analysis of National Institutes of Health Stroke Scale (NIHSS) scores and Hamilton Depression Scales (HAMD) to indicate post-stroke anxiety.

Considering that NIHSS and HAMD scores were independent risk factors of the PSA, we next determined the predictive cutoff threshold for NIHSS scores and HAMD scores using ROC curve analysis. A NIHSS cutoff score of 3.5 was able to differentiate patients with PSA from those without PSA, with a sensitivity of 66% and a specificity of 55%, resulting in an AUC of 0.642. By contrast, a HAMD cutoff score of 5.5 was able to differentiate patients with PSA from those without PSA, with a sensitivity of 79% and a specificity of 83%. AUC for PSA vs. non-PSA discrimination was 0.897 (95% CI 0.847–0.948, *P* < 0.001) (Figure 3).

## Discussion

The present study explored the incidence and predictors of PSA in elderly patients admitted to hospital following the COVID-19 outbreak and revealed a prevalence of 30.1% for PSA among elderly patients. We then compared epidemiological factors in 62 patients with PSA and 144 patients without PSA, and found that female gender, high NIHSS and HAMD scores were the independent factors for PSA by binary logistic regression.

PSA is a common neurological disorder that hinder patient rehabilitation (5). The COVID-19 outbreak has harmed psychosocial health, causing anxiety in the general population (22) and physicians (23). Compared to the 13.3% incidence of public anxiety in China during the COVID-19 outbreak (24), the prevalence of PSA with all ages in southern China has been reported between 1.2% and 27% before the COVID-19 pandemic (10, 12, 25). Our study found that 62/206 (30.1%) elderly patients developed PSA, which was higher than that prior to the COVID-19 outbreak but consistent with a recent report of 32% during the pandemic (26). We also identified fewer hospitalizations of elderly patients with ischemic stroke during the COVID-19 pandemic; this is likely a result of an unwillingness of patients to visit hospitals due to a fear of COVID-19 infection. Since the emergence of COVID-19, there has also been a stricter management of hospitalized patients including limitations on patient visitations from family members. Family members are unable to provide social support or emotional comfort directly to patients. Inpatients could therefore have also been psychologically impacted by these restrictive measures adopted by governments and societies.

We found that the incidence of PSA was higher in females than in males, probably due to the characteristic that female patients are more susceptible to social stress and other psychological factors (8, 27). A previous study assessing the symptoms of depression and anxiety in a neurology clinic also founded that female patients were more susceptible to anxiety than male (28). The single marital status (divorced or widowed) was not found as an independent risk factor for PSA, nonetheless, such patients were more prone to develop PSA by univariate analysis in the present study. It has been reported that lacking of companionship could reduce social support and guidance to patients, resulting in much severe anxiety and stroke mortality (29–31). We also found that more severe neurophysiological dysfunction at admission by the scores of NIHSS, mRS, MMSE, and HAMD in the PSA group than in the non-PSA group. However, after adjusting for confounding factors of gender, marital status, stroke severity and depression, the difference in the scores of mRS and MMSE between the two groups was no longer statistically significant, and only the scores of NIHSS and HAMD were associated with PSA by multivariate logistic regression analysis, in line with previous reports (32, 33). Furthermore, the cutoff threshold of NIHSS scores and HAMD scores were determined for independent predictors of PSA, with the result of 3.5 points (AUC of 0.64) and 5.5 points (AUC of 0.89), respectively. These insights may be helpful for clinicians to recognize PSA and provide more attention and support to such patients.

There were several limitations in the present study. First, this was a single-center retrospective study including inpatient data only. Second, the sample size was relatively small and no long-term follow-up of PSA was performed, possibly causing bias to some extent. Large-scale prospective studies are wanted to clarify these issues in the future.

## Data Availability Statement

The raw data supporting the conclusions of this article will be made available by the authors, without undue reservation.

## Ethics Statement

This retrospective study involving human participants was reviewed and approved by the local clinical trial committee of the First Affiliated Hospital of Sun Yat-Sen the identity University. All patient data were anonymized so that of the patients could not be ascertained in any way.

## Author Contributions

MY collected the data and wrote the paper. HL and YL conducted the data collection and undertook the statistical analysis. LL undertook the statistical analysis and provided revised suggestions. JY designed the study and revised the manuscript. All authors contributed to the article and approved the submitted version.

## Conflict of Interest

The authors declare that the research was conducted in the absence of any commercial or financial relationships that could be construed as a potential conflict of interest.
